# The role of emotion-related individual differences in enjoyment and masking smile judgment

**DOI:** 10.1186/s40359-023-01173-8

**Published:** 2023-04-25

**Authors:** Adèle Gallant, Annalie Pelot, Marie-Pier Mazerolle, René-Pierre Sonier, Annie Roy-Charland

**Affiliations:** 1 School of Psychology, Université de Moncton, 18 Avenue Antonine-Maillet, Moncton, NB E1A 3E9 Canada; 2 Department of Psychology, Laurentian University, Sudbury, ON Canada

**Keywords:** Masking smiles, Traces of negative emotions, Authenticity of smiles, Individual differences

## Abstract

**Background:**

While some research indicates that individuals can accurately judge smile authenticity of enjoyment and masking smile expressions, other research suggest modest judgment rates of masking smiles. The current study explored the role of emotion-related individual differences in the judgment of authenticity and recognition of negative emotions in enjoyment and masking smile expressions as a potential explanation for the differences observed.

**Methods:**

Specifically, Experiment 1 investigated the role of emotion contagion (Doherty in J Nonverbal Behav 21:131–154, 1997), emotion intelligence (Schutte et al. in Personality Individ Differ 25:167–177, 1998), and emotion regulation (Gratz and Roemer in J Psychopathol Behav Assess 26:41–54, 2004) in smile authenticity judgment and recognition of negative emotions in masking smiles. Experiment 2 investigated the role of state and trait anxiety (Spielberger et al. in Manual for the state-trait anxiety inventory, Consulting Psychologists Press, Palo Alto, 1983) in smile authenticity judgment and recognition of negative emotions in the same masking smiles. In both experiments, repeated measures ANOVAs were conducted for judgment of authenticity, probability of producing the expected response, for the detection of another emotion, and for emotion recognition. A series of correlations were also calculated between the proportion of expected responses of smile judgement and the scores on the different subscales.

**Results:**

Results of the smile judgment and recognition tasks were replicated in both studies, and echoed results from prior studies of masking smile judgment: participants rated enjoyment smiles as happier than the masking smiles and, of the masking smiles, participants responded “really happy” more often for the angry-eyes masking smiles and more often categorized fear masking smiles as “not really happy”.

**Conclusions:**

Overall, while the emotion-related individual differences used in our study seem to have an impact on recognition of basic emotions in the literature, our study suggest that these traits, except for emotional awareness, do not predict performances on the judgment of complex expressions such as masking smiles. These results provide further information regarding the factors that do and do not contribute to greater judgment of smile authenticity and recognition of negative emotions in masking smiles.

## Background

A smile possesses various necessary functions, most notably during social interactions. For instance, it can be an indication of happiness, affiliation, and cooperation, and can aid in the transmission of kindness and compassion [15, 18, 55]. While smile expressions may be produced during natural instances of felt happiness, there are times when the smile expression can be manipulated [13, 39]. For example, a smile can be simulated to mask a felt negative emotion (i.e., masking smile expressions, [14, 17]. However, masking a felt negative emotion with a smile expression is no simple task. An individual must not only activate the facial muscles associated with a smile but do so while also inhibiting the muscular activations associated with the felt negative emotion. The inhibition hypothesis proposes that attempting to mask a strongly felt emotion while purposely activating other muscles of the face leads to a leakage of the felt emotion, producing what has been termed in the literature as microexpressions [12, 13, 17, 44].

Microexpressions, are described as subtle facial muscular movements or full flashes of a dissimulated emotion [17]. Microexpressions are more likely to appear during strong emotional experiences, and occur in the upper or lower half of the face at once [43, 44]. A few studies have shown that individuals are often able to distinguish authenticity of enjoyment smiles from masking smiles. For instance, Perron et al. [42] investigated individuals’ abilities at judging enjoyment smiles and masking smiles containing traces of anger, sadness, fear and disgust. The results indicated that individuals were sensitive to the enjoyment and masking smiles as they more often classified the masking smiles as “not really happy” and more often reported the presence of another emotion for the masking smiles. Further, differences were observed as a function of the masked emotion as well as where the trace of negative emotion was presented (e.g., upper or lower face). For instance, masking smiles containing traces of anger in the eye area were rated as “really happy” more often than any of the other masking smiles, including the masking smile that contained traces of anger in the mouth area. However, identifying which negative emotion was being masked by the smiles proved to be a more difficult task, as shown from modest accuracy rates (between 20 and 50% accuracy). This has been the case with other studies of authenticity judgment and recognition of negative emotions in masking smile expressions as well (e.g., [23, 40].

In attempts to explain the differences in the judgment of authenticity and recognition of negative emotions in masking smiles, researchers have called upon the perceptual-attentional limitation hypothesis. This hypothesis posits that the accurate judgment of these expressions relies on the ability to attend to and perceive the necessary cues that distinguish the various expressions from each other [7, 41, 48, 49]. In other words, if the judgment of authenticity and recognition of negative emotions in masking smile expressions relied on perceptual-attentional processing, greater accuracy would have been expected to be associated with increased attention to the area of the face containing the trace of the negative emotion. For instance, for the angry-eyes masking smile, greater accuracy would be expected to be associated with greater attention to the eye area, as this is the area where the trace of the negative emotion was presented. However, Perron et al. [42] and Pelot et al. [40] employed eye-tracking to their studies of judgment of authenticity and recognition of negative emotions in masking smiles and found no relationship between perceptual-attentional processes and smile judgment or recognition. These results indicate that the perceptual-attentional limitations hypothesis does not consistently explain differences in the judgment of authenticity and recognition of negative emotions in these expressions. Thus, another source of explanation must be proposed to address these differences, which is the goal of the current paper.

Another path that could be worth exploring is that of individual differences. Many studies have explored the influence of individual differences in emotion recognition tasks of the basic emotions (anger, disgust, fear, happiness, sadness, and surprise). For instance, certain individual traits can hinder recognition of basic emotions. In fact, a study by Kahler et al. [29] found that those with high levels of trait hostility required a greater intensity level of the facial activations of the happiness emotion to correctly recognize the expression. Ferguson et al. [19] found that individuals with high trait anxiety were less accurate at correctly recognizing negative emotions. Studies have also shown that greater emotional dysregulation is related to greater difficulties in emotion recognition in various clinical populations [10, 30, 32, 56, 57]. On the other hand, other studies have suggested that other traits can help recognition, such as emotional intelligence [2, 50] and trait anxiety, in which high levels of this trait can help recognize the emotion of fear [53]. Thus, while some individual traits can hinder performance on recognition task, others can improve the performance, including those who are emotion related.

Furthermore, amongst individual differences, various emotion-related differences have also been documented as important in our perception and judgment of masking smiles and the authenticity of smiles. For instance, Pelot et al. [40] found significant negative correlations between difficulties in emotion regulation scores and accuracy at smile authenticity judgment of enjoyment and masking smiles in individuals from the general population with substance use disorders. However, this study found that the relationships between emotion regulation and smile judgment and emotion recognition were inconsistent and varied as a function of group, smile type, and subscale. It thus remains unclear how emotion regulation could impact the judgment of masking smiles and needs further investigation. On the other hand, more literature has suggested that emotion-related traits can help the recognition of these types of smiles. For instance, another form of anxiety, such as social anxiety, could have the potential to increase the ability to detect traces of negative emotions in masking smiles more accurately and rapidly [26]. Since anxiety has been found to both help and hinder recognition of basic emotions and smiles, it is worth testing systematically in the case of masking smiles considering this uncertainty. Additionally, high emotional contagion has also been found to positively influence the recognition of enjoyment (authentic,Duchenne and non-enjoyment smiles (non-authentic; non-Duchenne. In effect, Manera et al. [33] found that susceptibility for emotion contagion for negative emotions leads to better recognition of enjoyment smiles. However, accuracy decreased with increasing susceptibility to emotional contagion for positive emotions because it most often led to the categorization of non-enjoyment smiles as authentic. Thus, emotion-related traits seem to also have an influence in the judgment of enjoyment and non-enjoyment smiles, as well as smiles containing traces of negative emotions. However, this study explored the influence of this trait in enjoyment and non-enjoyment smiles that did not contain traces of negative emotions, as the case in masking smiles. Thus, the influence of emotional contagion on the judgment of a complex emotion such as a masking smile remains uncertain.

While several studies suggest that the emotion-related individual differences could contribute positively or negatively in emotion recognition, it remains unclear how some of these traits might influence judgment of more complexed expressions such as masking smiles. Thus, the goal of the current study is to explore the influence of such traits in the judgment of authenticity and recognition of negative emotions in enjoyment and masking smile expressions, in attempts to find other potential explanations for the differences observed in judgment. We conducted two experiments aimed at further investigating judgments of smile authenticity and recognition of negative emotions in masking smiles containing traces of fear, anger, sadness, and disgust. Specifically, the goal of Experiment 1 was to investigate the role of various emotion abilities (i.e., emotional contagion, emotional intelligence, and emotional regulation) in the accuracy of smile authenticity judgment and recognition of negative emotions in masking smile expressions. The aim of Experiment 2 was to examine the role of state and trait anxiety in the accuracy of smile authenticity judgment and recognition of negative emotions in masking smile expressions.

## Experiment 1

The current study investigated the role of various emotion-related abilities (i.e., emotional intelligence, emotional contagion, and emotional regulation) in the judgment of smile authenticity and recognition of negative emotions in masking smiles containing traces of fear, anger, disgust, and sadness. Further, we attempted to predict accuracy in smile authenticity judgment and recognition of negative emotions in masking smile expressions with each emotion ability variable (as in its own model and as a whole model) and examined relationships between accuracy at the judgment and recognition tasks and each emotion ability. The same judgment and recognition tasks used in Perron et al. [42] and Pelot et al. [40] were employed in the current study, except for measuring eye movements as they found that it was not a reliable indicator of task performance. As mentioned previously, emotional intelligence, emotional contagion, and emotional regulation have each been implicated in some way in our perception and recognition of emotions. Subsequently, we hypothesized that accuracy for smile authenticity judgment and recognition of negative emotions would be dependent on or related to these differences. Specifically, we hypothesized that based on previous literature (1) participants could distinguish the enjoyment smiles from the masking smiles, (2) differences in judgment of authenticity and recognition of negative emotions would be observed as a function of smile types (3) higher degrees of emotion contagion for negative emotions would be related to better accuracy of smile authenticity judgment and recognition of negative emotions for all types of smile, and (4) higher levels of emotional intelligence and emotion regulation would be related to an overall better performance of authenticity judgment and recognition of negative emotions.

## Method

### Participants

Seventy-seven individuals (52 females, 24 males and 1 other; *M*_AGE_ = 22.65) reporting normal or corrected to normal vision participated in the study. Sample size was selected based on similar studies. Power analyses were computed with G Power 3.1.9.7. With a medium effect size (e.g., 0.25), a sample of 20 participants is sufficient to obtain a power of 0.80 for the ANOVAs and a sample of 36 is sufficient for the regressions.

### Stimuli

**Fig. 1:**
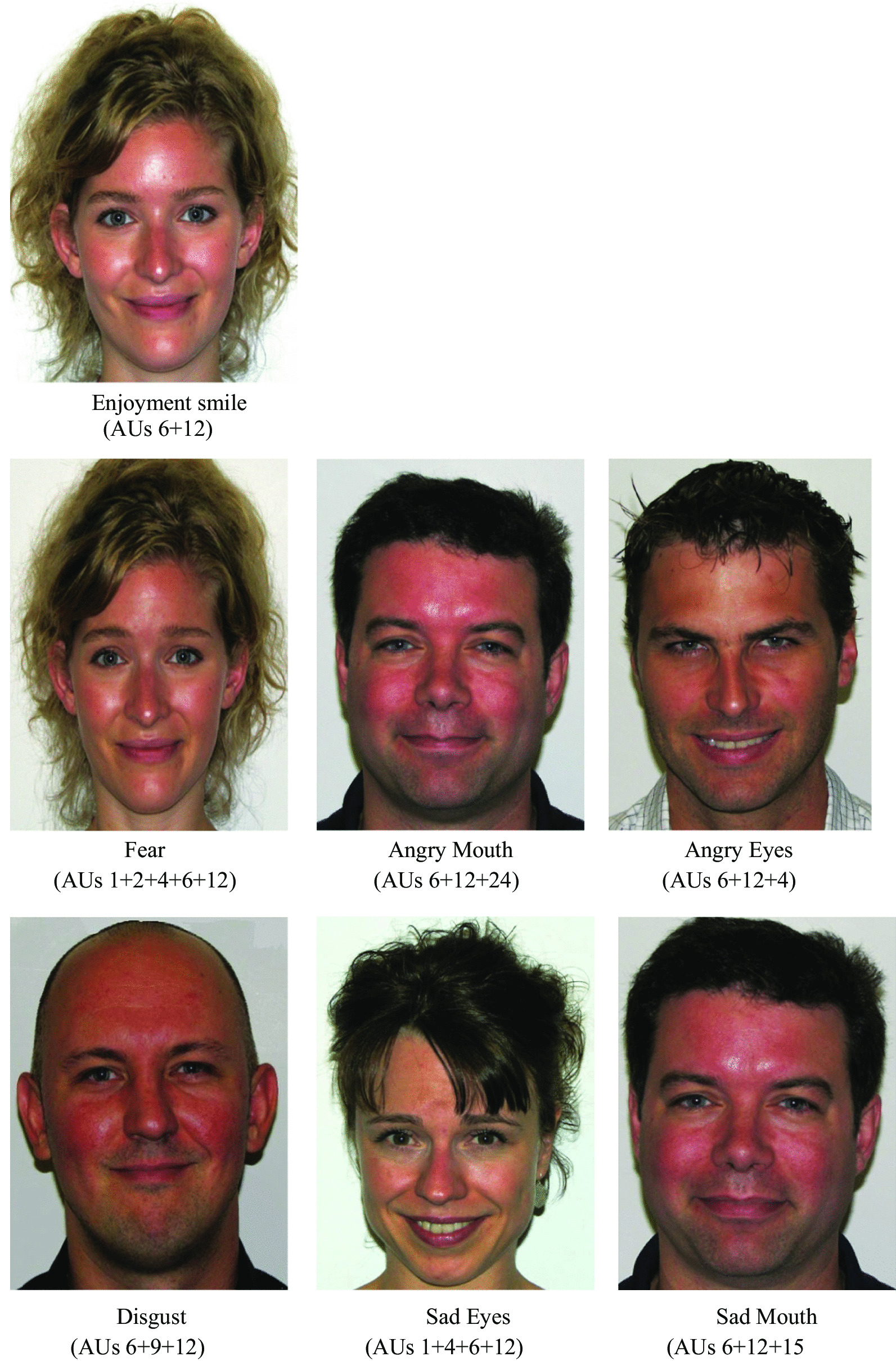
Sample of Stimuli. An example of the enjoyment smile is shown in the top panel while examples of the six masking smiles are shown in the lower panels. Stimuli were co-created by an author and the paper (ARC) and were initially used in [42]. ARC has co-ownership of the stimuli and has the right to use them. The masking smiles contained characteristics of the enjoyment smile and additional traces associated with fear, anger, sadness or disgust (the facial muscular activations corresponding to each emotional facial expression is presented below the image). Each trace of negative emotion was produced at an intensity level of B to reflect the subtlety of the activations within microexpressions

The stimuli employed within the current study were developed using the Facial Action Coding System (FACS; [16]. The 7 different types of smiles (see Fig. 1 for examples from the stimuli set) were initially used in Perron et al. [42], and consisted of one enjoyment smile and six masking smile expressions containing traces of fear, disgust, sadness in the eyes, sadness in the mouth, anger in the eyes, or anger in the mouth (see [42] for information regarding specific activations associated with each expression). Six different Caucasian individuals (3 males and 3 females) were recruited to produce the expressions. These individuals are referred to hereafter as *encoders*. Because some individuals had difficulty producing some of the expressions, the best 4 encoders per each of the smiles were selected for the final stimuli set. Thus, the complete stimuli set includes 28 smile expressions (1 enjoyment smile and 6 masking smiles) each produced by 4 individuals. Each of the smile expressions were produced and recorded under the guidance of a certified FACS coder. Reliability was established with 100% inter-rater agreeability following evaluation by two qualified FACS coders.

### Emotion-related individual differences questionnaires

All measures used in this study are open-access resources and are not under licence. All the measures used in this study were translated and previously validated.

*Emotional Contagion Scale* A French translated version of the Emotional Contagion Scale (ECS; [11] was used [46]. The ECS is a 15 item, self-report measure that examines the degree to which an individual is susceptible to be affected (i.e., mimicry tendency) by five basic emotions (i.e., love, happiness, fear, anger, and sadness).

*Emotional Intelligence Scale* A modified, French translated, version of the Emotional Intelligence Scale (EIS; [51] developed by Austin et al. [3] was used to assess emotional intelligence. This modified scale is a self-report questionnaire composed of 41 items that measure for domains,(1) regulation of emotion, (2) utilization of emotions and (3) appraisal of emotions.

*Difficulties in Emotion Regulation Scale* A reliable and validated French translated version of the Difficulties in Emotion Regulation Scale (DERS; [24] by [9] was used to assess emotion regulation abilities. The DERS is a 36-item self-report questionnaire that measures six areas of emotion regulation functioning: (1) nonacceptance of emotional response, (2) difficulties in adopting goal-directed behaviours, (3) difficulties in controlling impulsive behaviours, (4) lack of emotional awareness, (5) limited access to emotion regulation strategies and (6) lack of emotional identification or clarity.

*Apparatus* Participants view the photographs on a 21-inch VIEW-Sonic CRT monitor while stimuli are simultaneously presented on a second monitor for experimenter observation. The EyeLink II system (SR Research limited) was used a presentation tool, but eye movements were not recorded. The participant sits approximately sixty centimeters (cm) from their viewing computer screen.

### Procedure

**Fig. 2:**
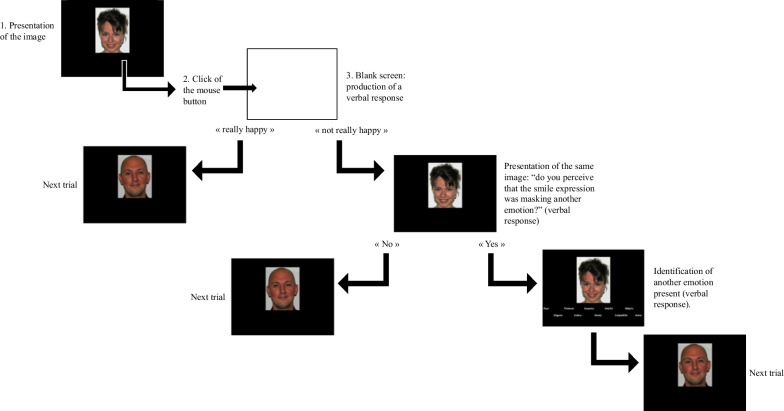
Example of the experimental procedure in Experiment 1 and 2

Participants took part in a single session of approximately 30 min in laboratory. judged 96 images, 48 images were characteristic of enjoyment smiles (4 encoders × 12 presentations) and 48 images were characteristic of masking smiles (4 encoders × 6 types of smiles × 2 presentations), presented in a random order, as defaulted with the EyeLink Experiment Builder software. The flow of the experimental procedure is illustrated at Fig. 2. All images were presented in the center of the screen on a white background. Once the image appeared on the screen, participants were instructed to click the mouse button when they were ready to provide their answer. At that moment, the image would disappear from the screen, and participants provided a verbal response, which the experimenter noted. Participants were instructed to respond “really happy” if they perceived the smile expression to be accurately representative of happiness, or “not really happy” if they perceived the smile expression was not accurately representative of happiness. If participants responded with “not really happy,” the same image would reappear on the screen where participants would be asked if they perceived that the smile expression was masking another emotion. If participants perceived another emotion was present, a list of 10 emotions (i.e., anger, fear, sadness, disgust, surprise, interest, guilt, shame, contempt and other) would appear below the image for the participant to choose from. After selecting an emotion from the list or providing their own verbal answer, the next trial would begin. Participants completed the three emotion ability questionnaires following the judgment task.

### Data analysis

Analyses were completed using R Core Team [45], and assumptions of each test were met. For all analyses, a 0.05 level of significance was adopted unless otherwise indicated. A within-subject analyses of variance (ANOVA’s) were used to examine differences in judgment (i.e., happiness, and detection of another emotion), and recognition (i.e., emotion identification) as a function of the 7 smile prototypes (enjoyment smile, disgust smile, angry-eyes smile, angry-mouth smile, sad-eyes smile, sad-mouth smile, and fear smile). An analysis was conducted comparing the probability of answering “really happy” among the 7 smile prototypes. An analysis was also conducted comparing the probability of producing the expected responses of “really happy” for the enjoyment smile and “not really happy” for the masking smiles. A third analysis was conducted comparing the probabilities of responding that another emotion was present within the smile, while another was computed to observe accuracy at identifying the masked negative emotions within the smile expressions. When Mauchly’s test of sphericity was significant for the ANOVA, the Huynh–Feldt correction was applied where epsilon (ε) was superior to 0.75 and the Greenhouse–Geisser correction was applied where epsilon (ε) was inferior to 0.75 [22]. Tukey HSD correction for repeated-measures analysis was used to decompose ANOVA main effects [27]. Correlations were computed to examine the relationships between accuracy at smile judgment and recognition of negative emotions as a function of EIS, ECS, and DERS questionnaires subscales scores. Finally, multiple regression analyses were conducted to explore the relationships between smile authenticity judgment and recognition of negative emotions (i.e., emotion identification) and total and subscale scores on the EIS, ECS, and DERS questionnaires. Data will be available upon request.

## Results

### Judgment of authenticity

**Fig. 3:**
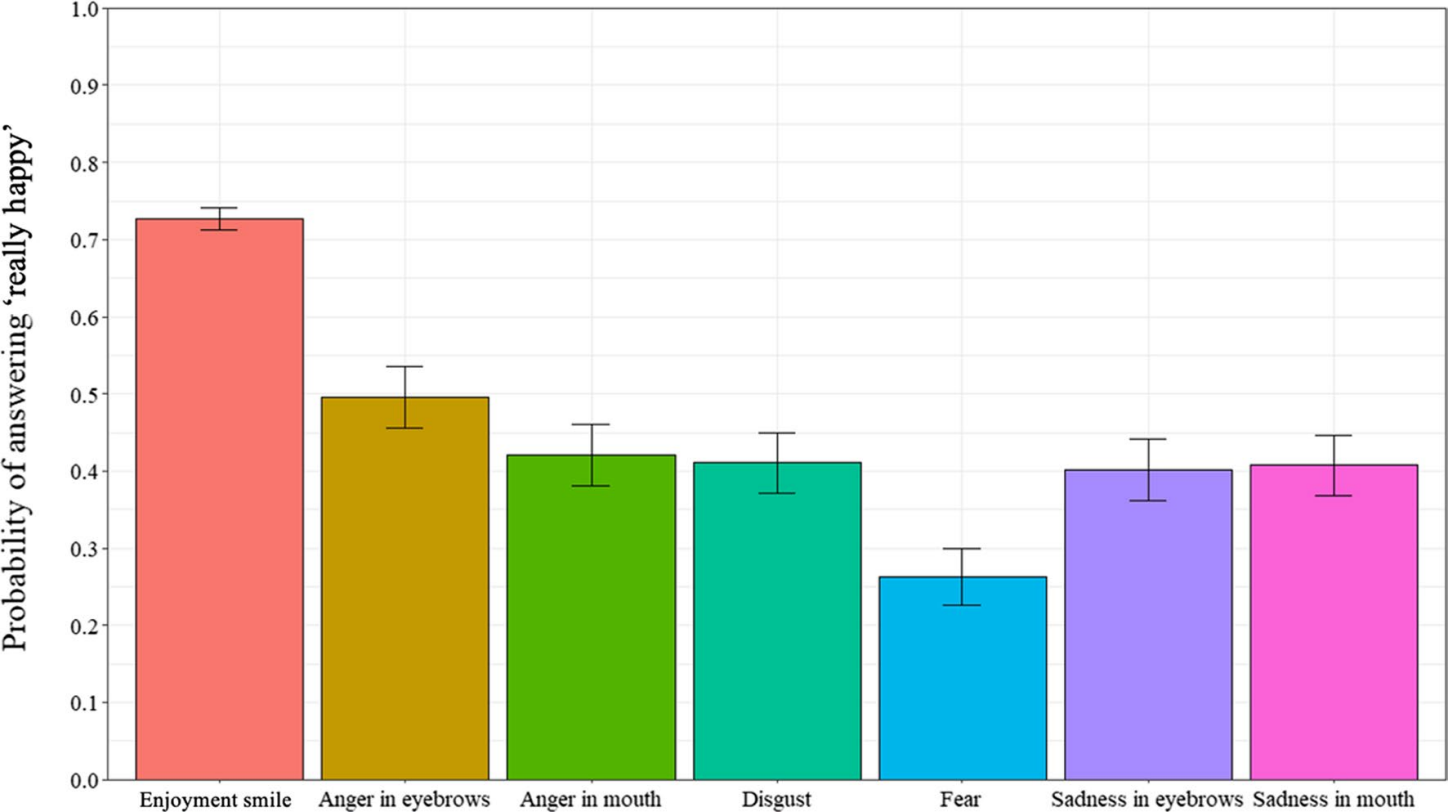
Probability of answering “really happy” as a function of types of smiles in Experiment 1. *Note* Error bars represent 95% within-participant confidence intervals computed according to Morey’s [36] procedure

An analysis was computed to examine the probability of responding “really happy” as a function of all 7 smile prototypes (Fig. 3). A repeated measures ANOVA examined the probability of responding “really happy” as a function of the 7 smile prototypes (enjoyment smile, disgust smile, angry-eyes smile, angry-mouth smile, sad-eyes smile, sad-mouth smile, and fear smile) (ε = 0.338). Results revealed a significant effect of smile prototype, *F*(4.07, 308.96) = 44.11, *p* < 0.001, *η*_*p*_^*2*^ = 0.37. Post-hoc tests revealed that participants answered “really happy” more often for the enjoyment smiles than all of the masking smile expressions (all *p*s < 0.001). Further, of the masking smile expressions, the angry-eyes smiles were rated more often as “really happy” than the sad-eyes smile (*p* = 0.027), while the fear smiles were rated as “really happy” least often (all *p*s < . 001).

### Probability of producing expected response

**Fig. 4:**
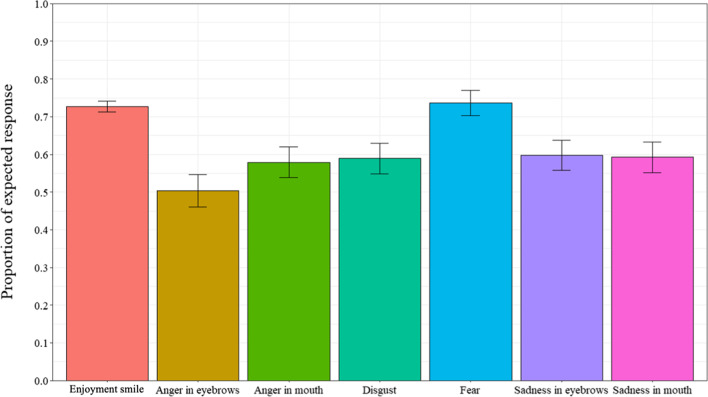
Proportion of expected responses as a function of types of smiles in Experiment 1. *Note* Error bars represent 95% within-participant confidence intervals computed according to Morey’s [36] procedure

Additional analyses were computed to determine if participants accurately judged enjoyment smiles as “really happy” and masking smiles as “not really happy”. A repeated measures ANOVA was performed between the proportion of expected responses and the 7 smile prototypes (enjoyment smile, disgust smile, angry-eyes smile, angry-mouth smile, sad-eyes smile, sad-mouth smile, and fear smile) (Fig. 4) (ε = 0.296). Results revealed a significant main effect for smile prototype, *F*(4.01,324.70) = 13.84, *p* < 0.001, *η*_*p*_^*2*^ = 0.15. Post-hoc tests revealed that participants produced the expected responses more often for the enjoyment smiles and the fear masking smiles (all *p*s < 0.001), with no significant differences between the two (*p* = 0.99). Further, participants produced the expected response more often for sad-eyes masking smiles than angry-eyes masking smiles (*p* = 0.046). Additional one sample t-tests were computed to determine if proportion of expected response for each type of smile was significantly different than chance (0.50 or 50%). Results showed that for all of the 7 smile prototypes, the proportion of expected response was significantly greater than chance, *t’*s (76) > 2.73, *p’*s < 0.009, Cohen’s *d’*s > 0.311, except for the angry-eyes masking smiles, *t*(76) = 0.17, *p* = 0.87, Cohen’s *d* = 0.019, which did not differ from chance.

### Detection of another emotion

**Fig. 5:**
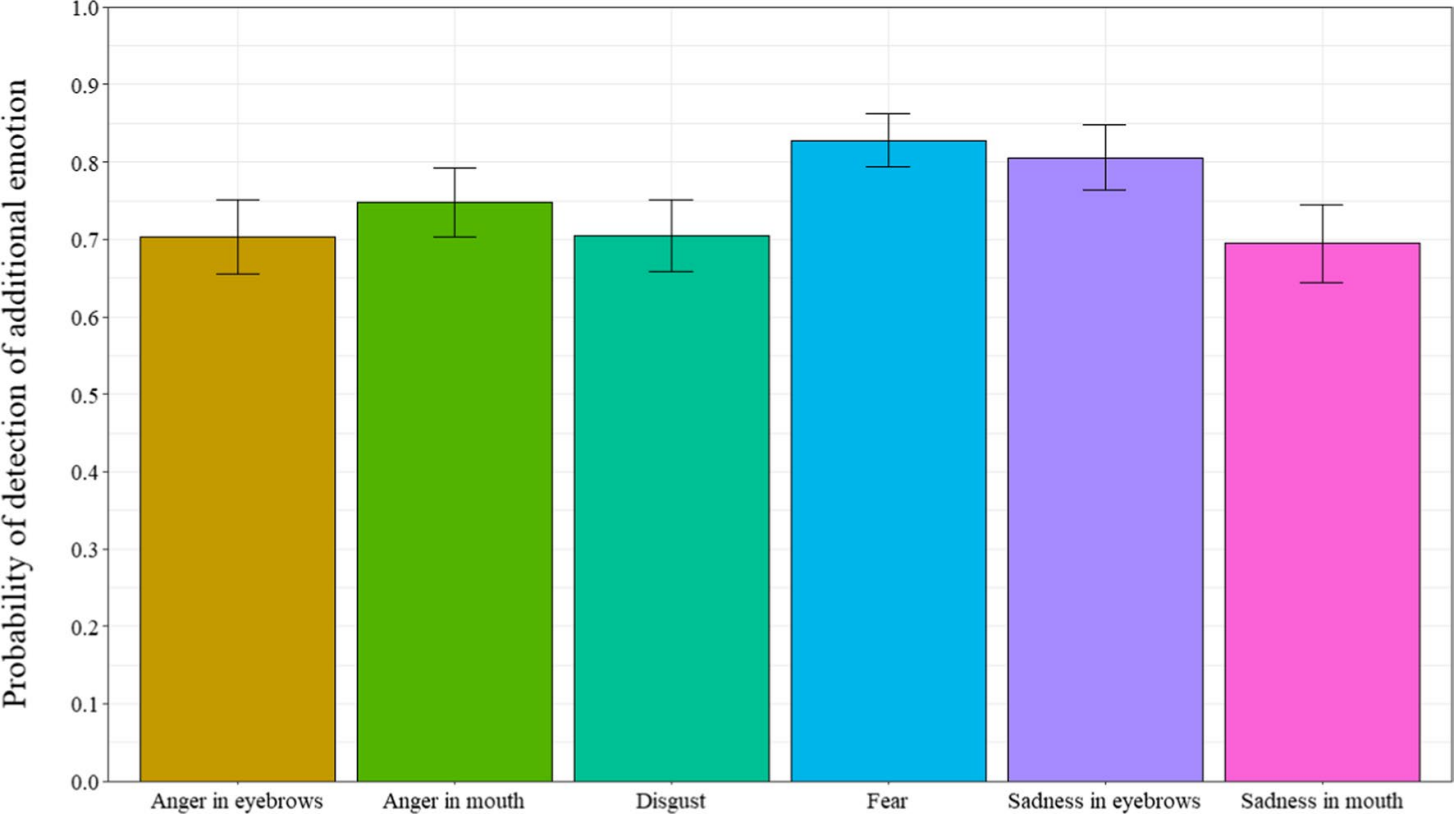
Probability of detecting an additional emotion as a function of types of smiles in Experiment 1. *Note* Error bars represent 95% within-participant confidence intervals computed according to Morey’s [36] procedure

When participants answered that smiles were “not really happy”, they were then asked if they could detect the presence of an additional emotion. The probability that participants reported the presence of an additional emotion was analyzed (Fig. 5). Only masking smile trials where participants responded with “not really happy” were analyzed (n = 2217, representing 30% of trials), as enjoyment smile trials were automatically incorrect when participants responded with “not really happy”. A repeated measures ANOVA was computed to examine the probability of detecting the presence of another emotion, with the 6 masking smile prototypes as a within-subject factor (ε = 0.625). Results revealed a significant main effect for smile prototype, *F*(4.34, 295.38) = 3.38, *p* < 0.007, *η*_*p*_^*2*^ = 0.047. Post-hoc tests revealed that participants detected the presence of an additional emotion more often for sad-eyes than anger-eyes, disgust, and sad-mouth masking smiles (respectively, *p* = 0.042; *p* = 0.016; *p* = 0.017). Participants detected the presence of another emotion significantly more often than chance levels for all masking smile prototypes, *t’*s (68) > 5.83, *p’s* < 0.001, Cohen’s *d’s* > 0.702.

### Emotion recognition

**Fig. 6:**
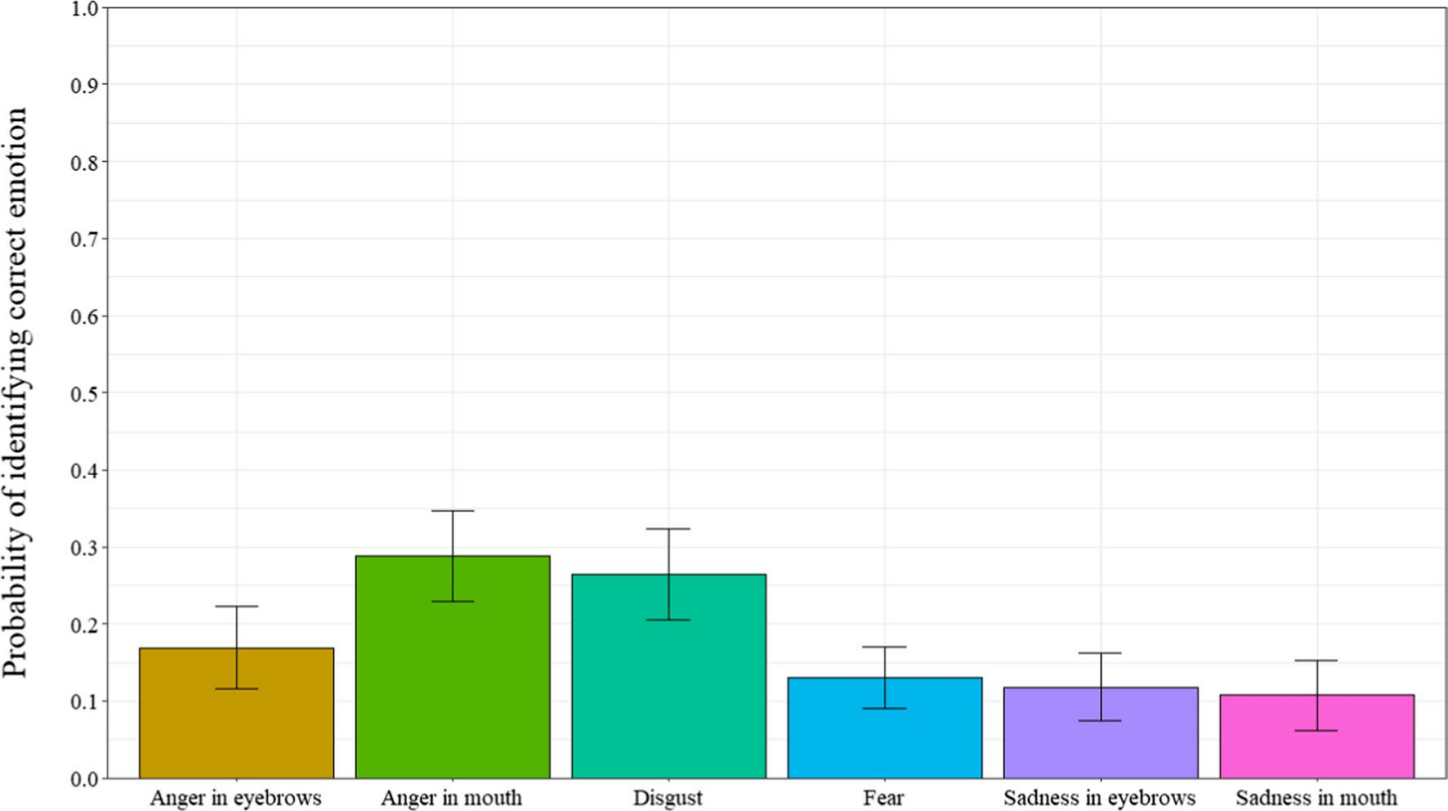
Probability of identifying correct emotion as a function of types of smiles in Experiment 1. *Note* Error bars represent 95% within-participant confidence intervals computed according to Morey’s [36] procedure

Once participants reported the presence of an additional emotion, they were asked to indicate from a list which emotion they detected. A repeated measures ANOVA was computed to observe the probability of identifying the correct emotion with the 6 masking smile prototypes as a within-subject factor (Fig. 6) (ε = 0.430). Results revealed a significant main effect for smile prototype, *F* (3.80, 220.14) = 7.99, *p* < 0.001, *η*_*p*_^*2*^ = 0.12. Post-hoc tests revealed that participants identified the correct emotion more often for the angry-mouth smile expressions than the angry-eyes, fear, sad-mouth, and sad-eyes smile expressions (respectively, *p* = 0.043; *p* < 0.001;* p* < 0.001; *p* = 0.002). Further, participants correctly identified the emotion more often for the disgust smile expressions compared to the fear, sad-mouth and sad-eyes smile expressions (respectively, *p* = 0.003; *p* < 0.001; *p* = 0.005). Results showed that participants are significantly better than chance levels (0.10) for identifying the angry-eyes, anger-mouth and disgust masking smiles, *t*s’(58) > 2.00, *p’*s < 0.05, Cohen’s *d’*s > 0.262. However, participants were not significantly different than chance for identifying the fear, sad-eyes and sad-mouth masking smiles, *t’*s (58) < 0.87, *p’*s > 0.38, Cohen’s *d’*s < 0.114.

### Correlations

Correlations between the probability of expected responses of smile judgment and the ECS subscale scores revealed no significant correlations for EC_Positive*, r* = − 0.04, *p* = 0.38 or EC_Negative, *r* = − 0.09, *p* = 0.22. Correlations between the proportion of expected responses of smile judgment and the EIS subscale scores revealed no significant correlations for EI_Optimism*, r* = − 0.001, *p* = 0.50, EI_Utilization, *r* = 0.13, *p* = 0.12, or EI_Appraisal*, r* = 0.09, *p* = 0.21. Correlations between the proportion of expected responses of smile judgment and the DERS subscale scores revealed a significant negative relationship for DERS_Awareness, *r* = -0.27, *p* = 0.01, but no significant relationships for DERS_Nonacceptance, *r* = − 0.09, *p* = 0.23, DERS_Goal, *r* = -0.03, *p* = 0.39, DERS_Impulse, *r* = − 0.04, *p* = 0.37, DERS_Strategies, *r* = − 0.16, *p* = 0.09, or DERS_Clarity, *r* = − 0.17, *p* = 0.06.

Correlations between the probability of identifying the correct negative emotions and the ECS subscale scores revealed no significant correlations for the enjoyment smiles and EC_Positive*, r* = 0.02, *p* = 0.43 or EC_Negative, *r* = − 0.07, *p* = *0.28,* or between the masking smiles and EC_Positive*, r* = − 0.08, *p* = 0.25 or EC_Negative, *r* = − 0.07, *p* = *0.27.* Correlations between the recognition of negative emotions and the EIS subscale scores revealed no significant correlations for the enjoyment smiles and EI_Optimism*, r* = 0.11, *p* = 0.16, EI_Utilization, *r* = 0.15, *p* = 0.10, or EI_Appraisal*, r* = 0.16, *p* = 0.09, or for the masking smiles and EI_Optimism*, r* = − 0.12, *p* = 0.15, EI_Utilization, *r* = 0.06, *p* = 0.31, or EI_Appraisal*, r* = − 0.02, *p* = 0.43. Correlations between accuracy at the recognition of negative emotions and the DERS subscale scores revealed no significant correlations between the enjoyment smiles and DERS_Awareness, *r* = − 0.12, *p* = 0.149, DERS_Nonacceptance, *r* = − 0.17, *p* = 0.07, DERS_Goal, *r* = 0.001, *p* = 0.50, DERS_Impulse, *r* = − 0.02, *p* = 0.42, DERS_Strategies, *r* = − 0.17, *p* = 0.07, or DERS_Clarity, *r* = − 0.12, *p* = 0.15. A significant moderate negative correlation was observed between the masking smiles and DERS_Awareness, *r* = − 0.30, *p* = 0.004, but not DERS_Nonacceptance, *r* = 05, *p* = 0.35, DERS_Goal, *r* = -0.05, *p* = 0.33, DERS_Impulse, *r* = − 0.04, *p* = 0.38, DERS_Strategies, *r* = − 0.07, *p* = 0.27, or DERS_Clarity, *r* = -0.15, *p* = 0.10***.***

### Predicting accurate smile authenticity judgment with the emotional abilities model

A standard multiple regression was computed with accuracy for expected judgment of happiness of all 7 smiles (enjoyment smile, disgust smile, angry-eyes smile, angry-mouth smile, sad-eyes smile, sad-mouth smile, and fear smile) as the predicted variable, while the emotional abilities model included the EC subscale scores, EI total and subscale scores, and DERS total and subscale scores as the predictor variables. A summary of all regression analyses can be found in Table 1. Results revealed that the emotional abilities model did not significantly predict accuracy for expected judgment of happiness, *F*(4,72) = 1.27, *p* = 0.29, *R*^*2*^ = 0.07, adjusted *R*^2^ = 0.01.

**Table 1:** Summary of regression analyses from Experiment 1

	B	SE B	*β*	*p*	*Sr*^*2*^
**Authenticity Judgement**					
ECS_P	.002	.003	.10	.61	.06
ECS_N	− .006	.007	− .17	.38	− .10
EIS	.003	.002	.21	.08	.04
EI_Optimism	− .001	.003	− .05	.71	− .04
EI_Utilization	.005	.005	.11	.35	.11
EI_Appraisal	.002	.004	.09	.54	.07
DERS	− .001	.001	− .09	.43	.01
DERS_Awareness	− .011	.005	− .26	.03	− .25
DERS_Nonacceptance	.000	.005	− .01	.96	− .01
DERS_Goal	.002	.006	.05	.72	.04
DERS_Impulse	.007	.007	.15	.35	.11
DERS_Strategies	− .009	.007	− .24	.22	− .14
DERS_Clarity	− .011	.011	− .13	.32	− .11
**Emotion Recognition**					
*Enjoyment Smiles*					
ECS_P	.005	.005	.20	.29	.12
ECS_N	− .011	.009	− .23	.23	− .14
EIS	.005	.002	.25	.03	.06
EI_Optimism	.002	.004	.06	.66	.05
EI_Utilization	.006	.007	.12	.34	.11
EI_Appraisal	.004	.005	.10	.48	.08
DERS	− .003	0.002	− .21	.07	.04
DERS_Awareness	− .007	.007	− .12	.30	− .12
DERS_Nonacceptance	− .007	.007	− .18	.29	− .12
DERS_Goal	.009	.008	.17	.29	.12
DERS_Impulse	.010	.010	.15	.34	.11
DERS_Strategies	− .011	.009	− .22	.26	− .13
DERS_Clarity	− .009	.015	− .07	.58	− .06
*Masking Smiles*					
ECS_P	− .001	.004	− .06	.77	− .03
ECS_N	− .001	.009	− .03	.89	− .02
EIS	− .001	0.001	− .12	.32	.01
EI_Optimism	− .005	.004	− .14	.31	− .12
EI_Utilization	.003	.007	.06	.63	.06
EI_Appraisal	.001	.005	.03	.81	.03
DERS	0.001	0.000	.19	.11	.04
DERS_Awareness	− .016	.007	− .27	.02	− .27
DERS_Nonacceptance	.006	.006	.17	.31	.12
DERS_Goal	− .005	.008	− .09	.56	− .07
DERS_Impulse	.004	.01	.07	.65	.05
DERS_Strategies	− .007	.009	− .14	.47	− .08
DERS_Clarity	− .014	.015	− .12	.34	− .11

### Predicting accurate emotion recognition with the emotional abilities model

A standard multiple regression was computed for both the Enjoyment smile condition and Masking smile condition with accuracy of emotion identification as the predicted variable while the emotional abilities model included the EC subscale scores, EI total and subscale scores, and DERS total and subscale scores as the predictor variables. Results revealed that the emotional abilities model did not significantly predict accuracy of emotion identification for the Enjoyment or Masking smiles, *Fs*(3,76) < 2.33, *p* > 0.06, *R*^*2*^ < 0.12, adjusted *R*^2^ < 0.07.

## Discussion

In this experiment, we further examined differences in the judgment of smile authenticity and recognition of negative emotions in masking smile expressions containing traces of fear, anger, sadness, and disgust. Specifically, we investigated the role of emotional contagion, emotional intelligence, and emotional regulation in the judgment of smile authenticity and recognition of negative emotions in masking smiles. Results revealed differences in the judgment of smile authenticity and recognition of negative emotions. Specifically, participants responded “really happy” more often for the enjoyment smiles and produced the expected response of “really happy” more often for the enjoyment smiles, indicating that they are sensitive to these smile expressions. These results are congruent with other studies of smile judgment which suggest that individuals are able to distinguish enjoyment smile expressions, even from a very young age [23, 40, 42]. Implications will be explored in the General Discussion.

Similar to other studies of masking smile judgment [40, 42], of the masking smile expressions, participants produced the expected response of “not really happy” more often for the masking smiles containing traces of fear. These results were produced at a probability greater than chance levels. Additionally, consistent with Perron et al. [42] and Pelot et al. [40], the results indicated that the ability to distinguish between the enjoyment and masking smiles varied not only as a function of the masked emotion, but also as a function of where the trace of negative emotion was presented (i.e., eyes or mouth area). For instance, the probability of producing the expected response was greater for the emotion of anger when the trace of anger was presented in the mouth area as opposed to the eye area. This further provides evidence to support the idea that not all masking smile expressions are equally perceived and judged.

Regarding the detection of the presence of another emotion, participants reported that they detected the presence of additional emotions for the sad-eyes masking smiles when compared to the angry-eyes, disgust, and sad-mouth masking smiles. Again, these results were produced at a probability greater than chance levels and indicate that although participants can correctly judge smile authenticity (i.e., correctly categorize “really happy” vs. “not really happy”), they experience difficulty judging whether they perceived the non-enjoyment smiles (i.e. masking smiles) to be masking another emotion. When participants did detect the presence of another emotion, they were best at identifying disgust than the fear, sad-mouth, and sad-eyes smile expressions.

In considering the relationships between the emotional abilities (i.e., emotional contagion, emotional intelligence, and emotional regulation), moderate negative relationships were observed for the masking smile expressions between accuracy at authenticity judgment and the DERS Awareness subscale, and between accuracy at emotion recognition and the DERS Awareness subscale. This is interesting when one considers that the Awareness subscale measures the lack of awareness one has towards emotions. Subsequently, it seems logical that the more aware participants were towards emotions (i.e., low Awareness subscale scores), the greater the accuracy in both the judgment of smile authenticity and recognition of negative emotions in masking smile expressions. In fact, it has been proposed that the ability to recognize emotions in others is closely related to our ability to self-regulate emotions [28, 31]. Nevertheless, it should be noted that out of all the correlations computed, only two of them were significant. Thus, it is probable that this is simply a reflection of a type I error and due to chance alone.

Interestingly, none of the emotion-related abilities models significantly predicted accuracy at the judgment of authenticity of the enjoyment or masking smile expressions, or accuracy at recognition of negative emotions in masking smile expressions. This indicates that these emotion-related abilities as a whole (i.e., emotion contagion, emotion intelligence, and emotion regulation) or individually, do not play a significant role in one’s ability to judge smile authenticity and recognize negative emotions in masking smiles. The lack of many significant relationships between the emotional abilities’ measures and the judgment of smile authenticity and recognition of negative emotions in masking smile expressions is both consistent and inconsistent with prior studies. For instance, the current results are similar to those from Pelot et al. [40] and Daros [10], which showed no consistent relationship between emotion regulation factors and participants’ judgments,indicating that emotion regulation plays less of a role in the judgment and recognition of enjoyment and masking smile expressions. Similarly, Austin [2] only found relationships between judgment of facial expressions of happiness and sadness and one aspect of emotion intelligence (i.e., the appraisal of emotions). Inconsistent with prior studies (e.g., [33], no relationship between emotion contagion and facial expression judgment and recognition were observed within the current study. However, a significant difference between the current study and prior studies are the types of stimuli used. Whereas these studies also adopted stimuli created from the FACS, they utilized various macroexpressions of emotions (i.e., full-faced presentation of basic the emotions), except for Pelot et al. [40] who used masking smiles. Aside from expressions of enjoyment, the current study further explored judgment of masking smile expressions, which comprise subtle traces of negative emotions. This rationale is explored further in the General Discussion section of the current paper.

## Experiment 2

In addition to various emotional abilities, another emotion-related individual difference that has been implicated in the judgment of facial expression perception and recognition is state and/or trait anxiety. Specifically, some research has found that trait anxiety is associated with an ability to recognize fearful facial expressions more accurately [53]. The current study attempted to further investigate the role of state and trait anxiety in smile authenticity judgment and recognition of negative emotions in masking smiles. Using the same judgment and recognition tasks as Experiment 1, Experiment 2 investigated the differences in accuracy of smile authenticity judgment and recognition of negative emotions in masking smiles as a function of state and trait anxiety. Further, we investigated the relationship between and attempted to predict accuracy at smile authenticity judgment and negative emotion recognition and state and trait anxiety. Similar to Experiment 1, we also hypothesized that (1) participants are able to distinguish the enjoyment smiles from the masking smiles (2) differences in judgment of authenticity and recognition of negative emotions will be observed as a function of the smile types. Further, it was hypothesized that (3) higher degrees of state and/or trait anxiety would be related to greater accuracy in the judgment of authenticity for the enjoyment and masking smiles, and (4) that trait anxiety would be related to greater recognition of masking smiles, or at least those containing traces of fear.

## Method

### Participants

Forty individuals (31 females and 9 males, *M*_AGE_ = 20.75) reporting normal to corrected to normal vision participated in this study. Sample size was selected based on the same criteria as Experiment 1.

### Materials and procedures

Materials and procedure are identical to those of Experiment 1, with one exception, after completing the smile judgment and recognition task, participants completed the French version of the State-Trait Anxiety Inventory (STAI; [21, 52] instead of the emotional abilities’ measures (EIS, ECS, and DERS questionnaires). The French version of the STAI has been found to be both a reliable and valid translated alternative for the STAI, which is a widely used measure that differentiates between the temporary “state” of anxiety and the more durable “trait” of anxiety. It can be used as a clinical tool to diagnose anxiety as well as to make the distinction between depression and anxiety more tangible [52]. The 40-item questionnaire is self-reported on a 4-point scale and includes 20 items measuring state anxiety and 20 items measuring trait anxiety. Internal consistency varies between 0.86 and 0.95 and test–retest reliability varies between 0.65 and 0.75 for a two-month interval [52].

### Data analyses

The exact same analyses as Experiment 1 were completed in the current study. Within-subject ANOVAs were used to examine differences in judgment (i.e., happiness and detection of another emotion) and recognition of negative emotions as a function of the 7 smile prototypes (enjoyment smile, disgust smile, angry-eyes smile, angry-mouth smile, sad-eyes smile, sad-mouth smile, and fear smile). Again, an analysis was conducted comparing the probability of answering “really happy” across the 7 smile prototypes and another analysis compared the probability of producing the expected responses of “really happy” for the enjoyment smile and “not really happy” for the masking smiles. Analyses also compared the probabilities of responding that another emotion was present and at identifying the masked negative emotions. Correlations were computed to examine the relationships between accuracy at smile judgment and recognition of negative emotions as a function of the scores on the STAI. Finally, multiple regression analyses were conducted to explore the relationships between smile judgment (i.e., judgment of happiness, and detection of another emotion) and recognition (i.e., emotion identification) and state and trait scores on the STAI.

## Results

For all analyses, a 0.05 level of significance was adopted. When Mauchly’s test of sphericity was significant for ANOVAs, the Huynh–Feldt correction was applied when epsilon (ε) was superior to 0.75 and the Greenhouse–Geisser correction was applied when epsilon (ε) was inferior to 0.75 [22]. Tukey HSD corrected for repeated-measures analysis was used to decompose ANOVA main effects [27].

### Judgement of authenticity

**Fig. 7:**
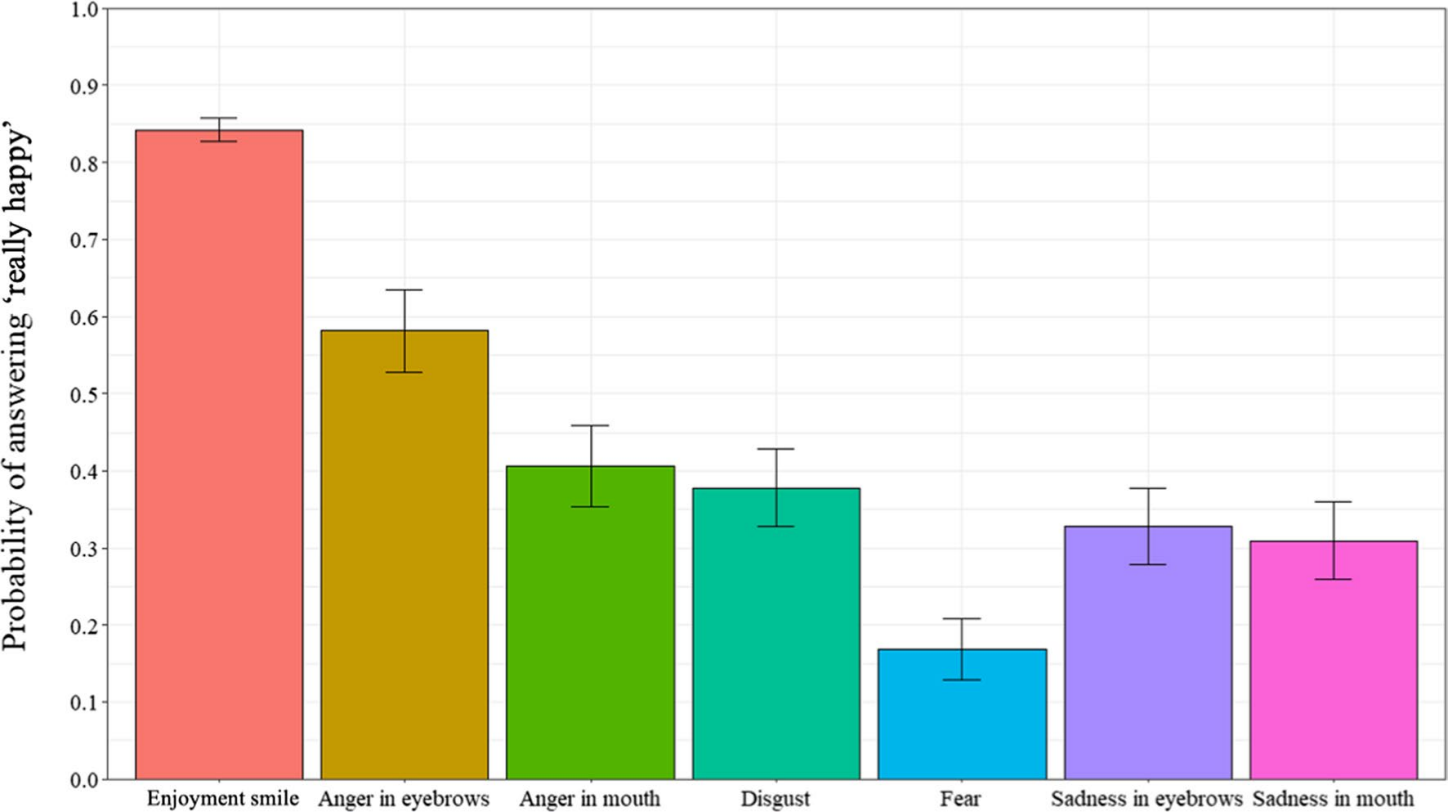
Probability of answering “really happy” as a function of types of smiles in Experiment 2. *Note* Error bars represent 95% within-participant confidence intervals computed according to Morey’s [36] procedure

Again, we began by analysing participants’ ratings of smiles as “really happy” or “not really happy”. Firstly, a paired sample t-test was computed on probability of answering “really happy” for the Enjoyment smile condition (*M* = 0.84, *SD* = 0.21) and all Masking smile conditions combined (*M* = 0.36, *SD* = 0.23). Results revealed a significant difference, *t*(39) = 15.36, *p* < 0.001, Cohen’s *d* = 2.429. In a second analysis, all of the 7 smiles were compared individually. A repeated measures analysis of variance, with types of smile (enjoyment smile, disgust smile, angry-eyes smile, angry-mouth smile, sad-eyes smile, sad-mouth smile, and fear smile) as a within-subject factor, was performed on the probability of selecting “really happy” (ε = 0.421). Results revealed a significant main effect, *F*(4.82,188.05) = 82.01, *p* < 0.001, *η*_*p*_^*2*^ = 0.68. As can be seen in Fig. 7, post-hoc tests revealed that participants answered “really happy” more often for enjoyment smiles than all of the masking smiles (all *p*s < 0.001). The angry-eyes smiles were rated as “really happy” more often than all of the other types of masking smiles (all *p*s < 0.001). Finally, smiles with traces of fear were rated “really happy” less often than all of the other smiles (all *p*s < 0.001).

### Probability of producing expected response

**Fig. 8:**
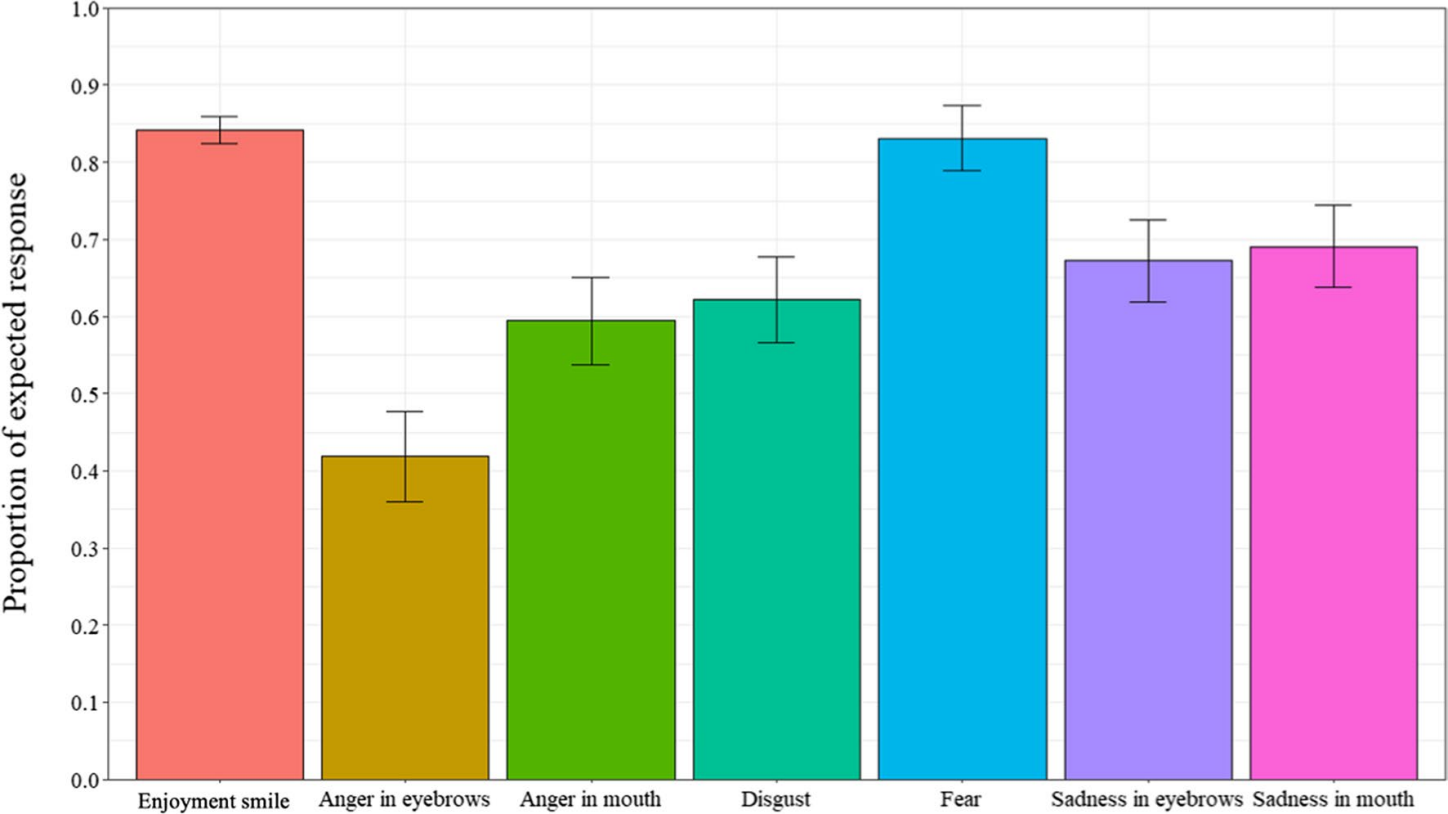
Proportion of expected responses as a function of types of smiles in Experiment 2. *Note* Error bars represent 95% within-participant confidence intervals computed according to Morey’s [36] procedure

Additional analyses were computed to determine if participants correctly judged if smiles were “really happy” or not according to the expected responses. Expected response for the Enjoyment smile condition was “really happy” while expected response for all the masking smiles was “not really happy”. A paired sample t-test was computed on proportion of expected response for the Enjoyment smile condition (*M* = 0.84, *SD* = 0.21) and all Masking smile conditions combined (*M* = 0.64, *SD* = 0.23). A repeated measure analysis of variance (ANOVA), with types of smile (enjoyment smile, disgust smile, angry-eyes smile, angry-mouth smile, sad-eyes smile, sad-mouth smile, and fear smile) as a within-subject factor, was performed on the proportion of expected responses (ε = 0.651). Results revealed a significant main effect, *F*(2.69,105.09) = 21.60, *p* < 0.001, *η*_*p*_^*2*^ = 0.37. As can be seen in Fig. 8, post-hoc tests revealed that participants produced the expected response more often for the Enjoyment smile and the Fear masking smile than all of the other types of smiles (all ps < 0.022), with no differences between the two (*p* = 1.0). Finally, participants selected the expected response least often for angry-brows smile than all of the other types of smiles (all *p*s < 0.001).

Additional one sample t-tests were computed to determine if proportion of expected response for each type of smile was significantly different than chance (0.50). Results show that for all of the smiles, proportion of expected response was significantly superior to chance, *t*s (39) > 2.30, *p*s < 0.03, Cohen’s *d*s > 0.363, except for the angry-brows smile, which was inferior to chance, *t*(39) = − 2.10, *p* = 0.04, Cohen’s *d* = 0.332.

### Detection of another emotion

**Fig. 9:**
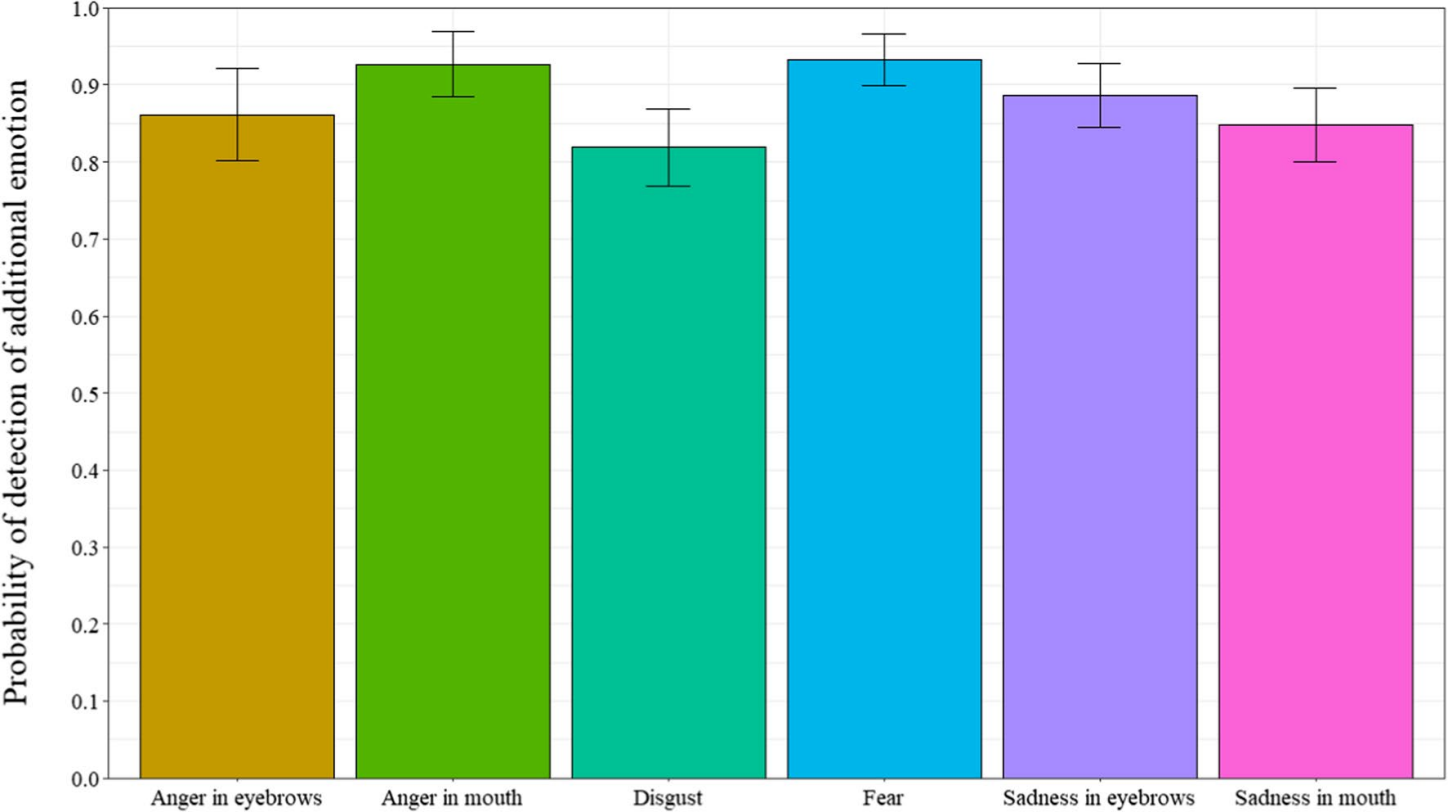
Probability of detecting an additional emotion as a function of types of smiles in Experiment 2. *Note* Error bars represent 95% within-participant confidence intervals computed according to Morey’s [36] procedure

When participants indicated that smiles were “not really happy”, they were then asked if they could detect the presence of another emotion, and if so, if they could identify the additional emotion. Thus, only trials where participants answered “not really happy” for the masking smiles conditions were analyzed (n = 1138, representing 30% of trials), as enjoyment smile trials were automatically incorrect when participants answered “not really happy”. We analyzed the probability that participants would detect an additional emotion in the smiles. A repeated measures analysis of variance, with types of smile (angry-eyes, angry-mouth, disgust, fear, sad-eyes, and sad-mouth) as a within-subject factor, performed on the detection of an additional emotion revealed a significant main effect, *F*(3.23,103.35) = 3.08, *p* = 0.01, *η*_*p*_^*2*^ = 0.09 (ε = 0.484). As can be seen in Fig. 9, post-hoc tests revealed that participants detected the presence of an additional emotion least often for the disgust smile than the angry-mouth and fear smiles (respectively, *p* = 0.023; *p* = 0.033). For every type of masking smile, participants detected the presence of another emotion significantly more often than chance, *t*s (32) > 7.49, *p*s < 0.001, Cohen’s *ds* > 1.304.

### Emotion recognition

**Fig. 10:**
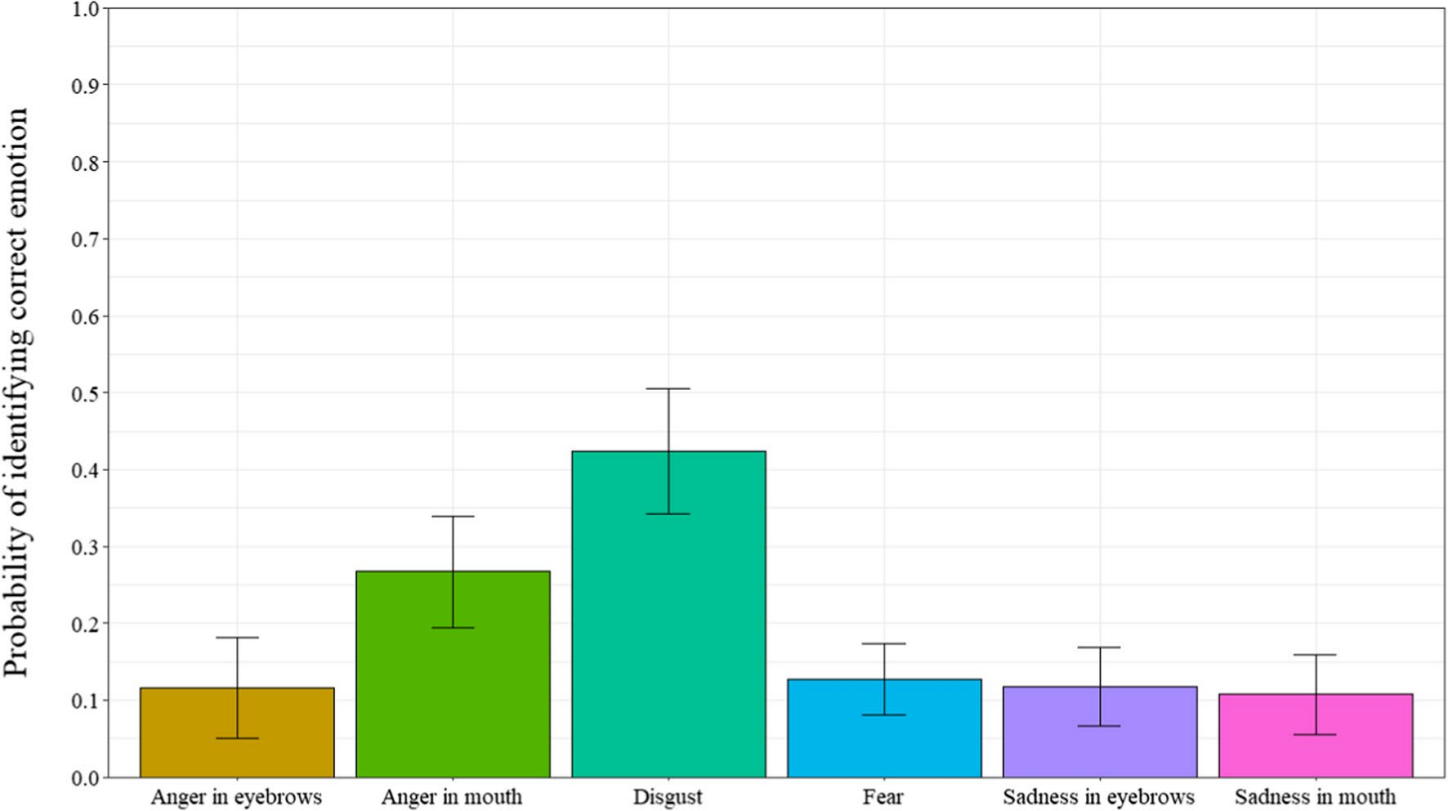
Probability of identifying correct emotion as a function of types of smiles in Experiment 2*. Note* Error bars represent 95% within-participant confidence intervals computed according to Morey’s [36] procedure

Once participants detected the presence of an additional emotion, they were asked to indicate from a list, which emotion they detected. A repeated measures ANOVA, with types of smile (angry-eyes, angry-mouth, disgust, fear, sad-eyes, and sad-mouth) as a within-subject factor was computed to examine the proportion of correct emotion identification. Expected response was the condition of the smile itself (angry-eyes, angry-mouth, disgust, fear, sad-eyes, and sad-mouth) (ε = 0.237). Results revealed a significant main effect of smile type, *F*(3.23,103.35) = 11.17, *p* < 0.001, *η*_*p*_^*2*^ = 0.26. As can be seen in Fig. 10, post-hoc tests revealed that participants identified the correct emotion more often for the angry-mouth smile than the angry-eyes smile, sad-mouth smile, and sad-eyes smile (respectively, *p* = 0.035;* p* = 0.004; *p* = 0.022) and more often for the disgust smile than all of the other types of smiles (all *p*s < 0.001), except angry-mouth (*p* = 0.133).

When comparing probabilities of identifying the correct emotion to chance (0.10), results show that participants are significantly better than chance for the angry-mouth and disgust smiles, *t*s(32) > 3.89, *p*s < 0.001, Cohen’s *d*s > 0.677, as can be seen in Fig. 10. However, for the angry-eyes, fear, sad-eyes and sad-mouth smiles participants responses were not significantly different than chance, *t*s (32) < 0.88, *p*s > 0.38, Cohen’s *d*s < 0.153.

### State-trait anxiety

State anxiety scores varied between 20 and 59 (*M* = 34.83) with 75% of scores ranging between 20 and 38.50, while trait anxiety scores varied between 21 and 59 (*M* = 39.75) with 75% of scores ranging between 21 and 45.25. A correlation between state and trait anxiety was computed. Results show a moderately positive correlation between both measures, *r* = 0.53, *t*(38) = 3.89,* p* < 0.001.

### Correlations

No significant correlations were observed between the probability of expected responses of smile judgment and state anxiety scores*, r* = 0.05, *p* = 0.18 or trait anxiety sores, *r* = 0.02, *p* = 0.43.

No significant correlations were observed between the probability of identifying the correct negative emotions and state anxiety scores for the enjoyment smiles*, r* = 0.08, *p* = 0.06 or the masking smiles *rs* > *0.01*, *ps* > 0.06. No significant correlations were observed between the recognition of negative emotions and trait anxiety scores for the enjoyment smiles*, r* = 0.11, *p* = 0.06 or the masking smiles *rs* > *0.06*, *ps* > 0.11.

### Predicting accurate smile authenticity judgment with state-trait anxiety

A standard multiple regression was computed with accuracy for expected judgment of happiness of smiles as the predicted variable and state and trait anxiety as predictor variables. A summary of all regression analyses can be found in Table 2. Results revealed that neither state or trait anxiety significantly predicted accuracy for expected judgment of happiness, *F*(2,37) = 0.92, *p* = 0.41, *R*^*2*^ = 0.05, adjusted *R*^2^ = 0.00.

**Table 2:** Summary of regression analyses from Experiment 2

	B	SE B	*β*	*p*	*Sr*^*2*^
**Authenticity Judgement**					
State anxiety	− .003	.002	− .26	.18	.05
Trait anxiety	.041	.002	.15	.43	.02
**Emotion Recognition**					
*Enjoyment Smiles*					
State anxiety	− .008	.004	− .35	.06	.08
Trait anxiety	.007	.004	.29	.11	.06
*Masking Smiles*					
Anger brows					
State anxiety	− .001	.002	− .14	.47	.01
Trait anxiety	− .000	.002	− .01	.96	.00
Anger mouth					
State anxiety	− .004	.003	− .28	.14	.06
Trait anxiety	.002	.003	− .14	.45	.01
Disgust					
State anxiety	− .004	.004	− .16	.40	.02
Trait anxiety	− .003	.004	− .14	.45	.01
Fear					
State anxiety	.003	.003	.19	.31	.03
Trait anxiety	− .002	.003	− .13	.49	.01
Sad brows					
State anxiety	.002	.002	.19	.30	.03
Trait anxiety	− .005	.002	− .35	.07	.09
Sad mouth					
State anxiety	.003	.002	.34	.06	.09
Trait anxiety	.000	.002	.03	.89	.00

### Predicting accuracy at emotion recognition with state-trait anxiety

A standard multiple regression was computed for accuracy on each smile condition (enjoyment smile vs. masking) individually, with accuracy for expected responses as the predicted variable and state and trait anxiety as predictor variables. Results revealed that for the enjoyment smiles or masking smiles, state and trait anxiety levels did not significantly predict accuracy for emotion recognition, *Fs*(2,37) < 2.79, *ps* > 0.07, *R*^*2*^ < 0.14, adjusted *R*^2^ < 0.09.

## Discussion

Experiment 2 further investigated the differences in the judgment of smile authenticity and recognition of negative emotions in masking smile expressions containing traces of fear, anger, sadness, and disgust. In terms of accuracy and in the detection of another emotion, the results of this experiment replicated those observed in Experiment 1. While the General Discussion discusses similarities and implications in the two experiences, the present discussion will focus solely on the distinctions observed in Experiment 2.

In considering the relationships between state and/or trait anxiety and smile authenticity judgment and emotion recognition in masking smiles, no significant relationships were observed for any of the smile expressions. Interestingly, neither state nor trait anxiety models significantly predicted accuracy at the judgment of authenticity of the smiles, or accuracy in recognition of the traces of negative emotions. Therefore, while there is good evidence suggesting that anxiety modulates other aspects of threat-related facial expressions, such as allocation of attention [4], the process of making judgments regarding smile authenticity and the recognition of masked negative emotions may not be modulated by state nor trait anxiety, unlike in the case of recognition of basic emotions [19, 53]. These findings are incongruent with early cognitive theories of emotional disorders [5, 6] which proposed that anxiety would be associated with biases that favour the processing of emotion-related stimuli across all domains of information processing. However, these results lend support to other studies that have found no relationships between trait anxiety, as measured by the STAI, and emotional facial expression judgment and recognition [8].

In sum, Experiment 2 revealed that the emotion-related individual difference of trait and state anxiety, as measured by the STAI, were not related to or able to predict accuracy at smile authenticity judgment or negative emotion identification in masking smiles. Analyses demonstrated that results were similar to those of previous studies by establishing a base effect consistent with that of Perron et al. [42] and Pelot et al. [40], thus the lack of relation between anxiety and the accuracy at smile authenticity judgment and recognition of negative emotions in masking smiles is most likely not due to unexpected results.

## General discussion

The overall goals of the current paper were to use two independent experiments to further contribute to current literature regarding the differences in the judgment of smile authenticity and recognition of negative emotions in masking smiles containing traces of fear, anger, sadness, and disgust. Further, the role of emotional abilities (i.e., emotional contagion, emotional intelligence, and emotional regulation) and state and trait anxiety in smile authenticity judgment and recognition of negative emotions in masking smiles were explored. Studies suggest that individuals can recognize smile authenticity at a fairly high rate (e.g., [40, 42]. However, these same studies have shown that individuals experience difficulty recognizing the presence of emotions within masking smiles as well as in identifying the masked negative emotions. The smile judgment and negative emotion recognition tasks in both Experiments 1 and 2 revealed results that resembled those from prior studies [23, 40, 42], and subsequently reinforces the notion that individuals are generally capable of determining enjoyment from non-enjoyment smiles, as shown from their ability to accurately categorize the enjoyment expressions as “really happy”.

Regarding the masking smiles, judgments of authenticity and recognition of the negative emotions differed according to the negative emotion and where the traces of negative emotion was presented, indicating that some of the masking smile expressions are more difficult to accurately judge than others. Results are consistent with those from prior studies which have found that participants’ ability to recognize and identify the negative emotions are modest, only reaching chance levels for one or two of the masking smiles. In fact, these tasks may rely more heavily upon other factors such as the context of the interaction and the observable temporal dynamics of the facial muscles of the smile expression, factors that have each been shown to increase accuracy in the differentiation of emotional facial expressions [25, 35, 47]. Additionally, in both Experiment 1 and 2, results indicate that of the masking smile expressions, participants seemed to be most sensitive to the smiles containing traces of fear. In fact, they produced the expected response of “not really happy” more often for the masking smiles containing traces of this emotion, and so, at above chance levels. The fact that individuals were most sensitive to the smile expressions containing traces of fear is not surprising when considering some theorists of evolved fear modules suggest that we as humans have developed behavioural, neural, and mental systems that are preferentially activated by fear-related stimuli (e.g., smiles containing traces of fear), thus allowing for the quick response towards perceived danger or threat within the environment [1, 37, 38].

Furthermore, regarding the detection of another emotion, participants detected the presence of additional emotions least often for masking smiles with traces of disgust, when compared to the angry-mouth and fear masking smiles. However, when tasked with identifying the masked emotion, participants were more often correct for the angry-mouth and disgust smiles. Interestingly, the disgust expression is the only expression that contains an activation associated with the nose (i.e., AU9, the Nose Wrinkler), which could suggest that this activation can serve as a useful cue when trying to detect the trace of a negative emotion. Nevertheless, these results further highlight the difficulty individuals experience with regards to recognizing and identifying the negative emotions within masking smile expressions. The results are similar as the one in Experiment 1, whereby the judgments of smile authenticity and recognition of negative emotions vary as a function of both the hidden negative emotion and where the trace of negative emotion is presented. (i.e., eyes or mouth).

There has been some support for the role of various emotion-related individual differences, such as emotional contagion, emotional intelligence, emotional regulation [33, 40] and trait anxiety [19, 53], in the recognition of emotions. Within the current study, relationships were only observed between one aspect of emotion regulation and smile judgment and emotion recognition. Specifically, the emotion awareness subscale of the DERS was related to smile judgment and emotion recognition of masking smiles only. There was no evidence to support the idea that these emotion-related individual differences as a whole predict either accuracy at the authenticity judgment task or ability to accurately recognize a negative emotion present in complex emotions such as masking smiles. Results of the current study reveal similar findings to Pelot et al. [40] who examined the role of emotional-related individual differences with a population with substance use disorders on the judgment of masking smiles. Like our study, they did not find significant relationships between emotion regulation abilities and judgment of enjoyment and masking smile emotions. These results are also similar to studies examining the recognition of macroexpressions of emotions (i.e., happiness, sadness) who did not find significant relationships between emotion regulation abilities and aspects of emotional intelligence and judgment and recognition (e.g., [2]. However, the results differ from Austin [2] who found a strong relationship between one aspect of emotional intelligence (i.e., appraisal of emotions) and judgment of emotions, and Manera et al. [33], who found that accuracy in a smile authenticity judgement task improved with increasing susceptibility to emotional contagion for negative emotions and decreased with increasing susceptibility to emotional contagion for positive emotions.

The differences observed across studies may be due to factors such as differences in the stimuli used within the three studies. For instance, these prior studies used enjoyment smile expressions that contained the activation of AU6 and AU12 (i.e., “authentic”, Duchenne smile), similar to our study. However, in these other studies, judgments were made against non-enjoyment smile expressions or macroexpressions of negative emotions (e.g., sadness, anger, fear) that did not contain AU6 or AU12 activations. Within the current study, the non-enjoyment smiles were masking smile expressions, which contain the AU6 and AU12 activations in addition to activations associated with the negative emotions. Therefore, the discrimination between the enjoyment and non-enjoyment smiles may have been more difficult within our study when compared to Manera et al. [33] and Austin et al. [3] as the non-enjoyment smile expressions within our study (i.e., masking smiles) still contain all of the activations associated with an enjoyment smile expression with very subtle nuances between the expressions. Indeed, research indicates an ease and universality in the judgment and recognition of macroexpressions of emotions that is not observed in the judgment of microexpressions of emotion [34]. Future studies might benefit from exploring the role of and differences between emotion related abilities across both micro-expressions and macro-expressions of emotion.

As it relates to the role of anxiety, findings from our study support those found in Cooper et al. [8] and further indicate that there may be no relationship between anxiety and emotional facial expression judgment and recognition but differ from other studies who found that anxiety could hinder this performance (e.g.., [19]. In fact, our results extend on previous studies which utilize macroexpressions of emotion and indicate that anxiety may not play a significant role in the judgment and recognition of some micro-expressions, specifically, masking smile expressions. Nevertheless, future research should continue to investigate factors that would contribute to greater accuracy at the recognition and identification of negative emotions within masking smile expressions as being able to accurately judge and recognized masking smile expressions would have adaptive value in communicatory interactions.

### Limitations

It is important to note the limitations to the current studies. For instance, we used static images as to the best of our knowledge, no bank of dynamic masking smile expressions exists just yet. Subsequently the use of static images could have had an impact on our results because the movement of the hidden negative emotion (microexpressions) in dynamic stimuli may better attract the attention of the participant and contribute to the recognition of the negative emotions [25, 35, 47]. Future studies should explore this possibility in order to determine whether dynamic stimuli can have an impact in the task of smile authenticity and recognition of negative emotions in masking smiles. Specifically, they could focus on creating new dynamic stimuli of masking smile expression, with evoked negative emotions that are intentionally masked, which would then be followed by validation of certified FACS coders. The results of such studies would further increase our ability to draw conclusions with real-world applications of enjoyment and masking smile judgment and recognition. Investigating the same tasks used within the current study but with the proposed dynamic stimuli might generate effects that were not visible in this study, thus adding valuable information on the emotion-related individual differences and their impact on masking smile recognition. Another limitation to both studies was the female dominant sample. However, literature has not provided conclusive support for gender effects of facial expression judgment and recognition in adult populations e.g. [20, 54]. Nevertheless, attempts should be made to include an equal female-male sample in future studies of enjoyment and masking smile judgment and recognition to examine possible gender effects.

## Conclusion

The present project evaluated the role of various emotion-related individual differences in the accurate ability to judge smile authenticity and recognize negative emotions in enjoyment and masking smiles containing traces of fear, sadness, disgust, and anger. The results showed that individuals were sensitive to the enjoyment and masking smile expressions. Variations in judgment were observed between the negative emotions with best accuracy shown for smiles containing traces of fear and worst for smiles containing traces of anger in the eye area. However, when the presence of a negative emotion was reported, participants were less accurate in identifying fear and more accurate at identifying disgust. Nevertheless, the recognition rates of these emotions were modest. Overall, except for emotional awareness, a skill involved in emotion regulation, the emotion-related individual differences did not account for differences in the judgment and recognition abilities in the present study. Traits that have been found to have a significant influence on judgment of basic emotions and in the judgment of enjoyment smiles seem not to be related to the differences observed in the case of complex expressions such as masking smiles. These results provide further information regarding the factors that do and do not contribute to greater judgment of smile authenticity and recognition of negative emotions in masking smiles. The importance in furthering research in the field of emotional facial expression recognition lie in the possibilities of understanding a subtype of characteristics or abilities that could potentially be developed to understand manners in which individuals could be trained in improving this skill.

## Data Availability

The datasets used during the current study is available upon request to the corresponding author.

## Abbreviations

DERS: Difficulties in emotion regulation scale
ECS: Emotional contagion scale
EIS: Emotional intelligence scale
EC: Emotional contagion
EI: Emotional intelligence
FACS: Facial action coding system
STAI: State-trait anxiety inventory
